# RNA-Based Therapeutics: Current Developments in Targeted Molecular Therapy of Triple-Negative Breast Cancer

**DOI:** 10.3390/pharmaceutics13101694

**Published:** 2021-10-15

**Authors:** Sakib Haque, Kiri Cook, Gaurav Sahay, Conroy Sun

**Affiliations:** 1 College of Pharmacy, Oregon State University, Corvallis, OR 97331, USA; haques@oregonstate.edu (S.H.); sahay@ohsu.edu (G.S.); 2 Department of Radiation Medicine, Oregon Health & Science University, Portland, OR 97239, USA; cooki@ohsu.edu

**Keywords:** triple-negative breast cancer, RNA interference, small interfering RNA, microRNA, messenger RNA, gene silencing, nanotechnology

## Abstract

Triple-negative breast cancer (TNBC) is a highly heterogeneous and aggressive cancer that has the highest mortality rate out of all breast cancer subtypes. Conventional clinical treatments targeting ER, PR, and HER2 receptors have been unsuccessful in the treatment of TNBC, which has led to various research efforts in developing new strategies to treat TNBC. Targeted molecular therapy of TNBC utilizes knowledge of key molecular signatures of TNBC that can be effectively modulated to produce a positive therapeutic response. Correspondingly, RNA-based therapeutics represent a novel tool in oncology with their ability to alter intrinsic cancer pathways that contribute to poor patient prognosis. Current RNA-based therapeutics exist as two major areas of investigation—RNA interference (RNAi) and RNA nanotherapy, where RNAi utilizes principles of gene silencing, and RNA nanotherapy utilizes RNA-derived nanoparticles to deliver chemotherapeutics to target cells. RNAi can be further classified as therapeutics utilizing either small interfering RNA (siRNA) or microRNA (miRNA). As the broader field of gene therapy has advanced significantly in recent years, so too have efforts in the development of effective RNA-based therapeutic strategies for treating aggressive cancers, including TNBC. This review will summarize key advances in targeted molecular therapy of TNBC, describing current trends in treatment using RNAi, combination therapies, and recent efforts in RNA immunotherapy, utilizing messenger RNA (mRNA) in the development of cancer vaccines.

## 1. Introduction

### 1.1. Triple-Negative Breast Cancer

#### 1.1.1. Clinical Significance of TNBC

Breast cancer (BCa) is one of the most frequently diagnosed cancers in women and presents as a major public health concern worldwide. In 2018, there were an estimated 2.1 million new cases identified around the world, of which approximately 627,000 cases resulted in death [1]. In general, BCa represents a highly diverse collection of malignancies, of which there are five intrinsic subtypes, identified through genomic studies (basal-like, HER2-enriched, claudin-low, luminal A, and luminal B), and a normal breast-like group [2]. Triple-negative breast cancer (TNBC) is a highly heterogeneous disease, representing approximately 15% to 20% of all BCas, and is considered to be the most aggressive form [3]. It is most commonly associated with the basal-like and claudin-low intrinsic molecular subtypes, where approximately 49 and 30 percent of TNBCs are classified as each, respectively [4]. TNBC has a poorer prognosis as compared to other BCa subtypes with a correspondingly significant mortality rate, recorded to be the highest of all subtypes within the first 3 to 5 years of diagnosis [5,6]. The lack of effective treatments developed for TNBC is a significant unmet clinical need in oncology. Most cancer therapeutics, including endocrine and chemotherapies, target one of three cell surface receptors found on BCa cells that drive their cell growth—estrogen (ER), progesterone (PR), and human epidermal growth factor 2 (HER2) receptors; however, TNBC cells are characterized by the absence of these. Due to this, standard therapeutics that have been successful in the targeted treatment of BCa are typically ineffective for TNBC and are unable to block cell proliferation. With this challenge in mind, there have been significant research efforts dedicated to implementing new therapies that can target TNBC cells. These therapies were principally founded on discovering specific molecular targets of TNBC and further elucidating the molecular pathogenesis of this highly heterogeneous and aggressive cancer.

#### 1.1.2. Molecular Pathogenesis and Description of Key Molecular Targets

Gene expression profiling studies have revealed seven distinct subgroups under which TNBCs can be classified: basal-like 1 (BL1), basal-like 2 (BL2), mesenchymal-like (M), mesenchymal stem-like (MSL), luminal androgen receptor (LAR), immunomodulatory (IM), and unstable (UNS) [7,8,9]. Each molecular subtype has differential expression of specific genes implicated in distinct signaling pathways, which dictate their principal modes of pathogenesis. The BL1 and BL2 subtypes, representing approximately 10–18% and 11–20% of TNBCs, respectively, show elevated expression of DNA-damage response and cell division genes, as well as genes involved in controlling cell proliferation [8]. BL2 subtypes also show upregulation of genes involved in glycolysis, gluconeogenesis, and increased growth factor signaling. The UNS subtype, representing approximately 10–14% of TNBCs, is characteristically like the BL1 and BL2 subtypes in that it also shows an upregulated expression of genes involved in DNA-damage response and control of cellular proliferation [10]. The IM subtype represents approximately 20% of TNBCs and is characterized by increased immune signaling genes, including those involved in cytokine signaling and antigen processing and presentation, and the M and MSL subtypes, representing approximately 20% and 7–10% of TNBCs, respectively, show elevated expression of genes involved in cell motility, differentiation, and the epithelial–mesenchymal transition [10]. The M subtype is also characterized by the increased expression of genes involved in cell proliferation, while the MSL subtype shows increased growth factor signaling and, uniquely, an upregulation of angiogenic genes. Lastly, the LAR subtype represents approximately 10% of TNBCs and is characterized by increased gene expression in three distinct signaling pathways, including androgen and estrogen metabolism, steroid synthesis, and porphyrin metabolism [10,11].

The molecular classification of TNBC has progressed significantly in the past few years, paving the way for more accurate molecular characterization of TNBC tumors. The approach used above is based on the Lehmann subtype classification, which has evolved into the Burstein four subtype classification. This system divides the former six subtypes into basal-like immunosuppressed (BLIS), basal-like immuno-activated (BLIA), LAR, and MES (mesenchymal-like). The BLIS subtype “expresses the immunosuppressive molecule V-set domain-containing T-cell activation inhibitor 1 (VTCN1)”, while the BLIA subtype “expresses STAT signal transduction molecules and releasing cytokines” [12]. The Burstein classification was then further modified into the Fudan University Shanghai Cancer Center (FUSCC) classification system, which also utilizes four criteria—IM, LAR, MES, and BLIS [12]. Evidently, all these classification systems can be used to effectively characterize different TNBC subtypes. However, it is important to recognize that while there are certain similarities between the systems, including the overlap between the MSL and MES subtypes, IM with BL1 and BLIA subtypes, and M with the BLIS subtype, the different classification criteria used by each should be both considered individually and used in tandem for diagnostic purposes in the clinical environment for greater accuracy in characterizing TNBC tumors [13].

Within each of the Lehmann subtypes, and their respective implicated signaling pathways, there are important molecular markers present that can be targeted, offering potential therapeutic options. For example, the MSL subtype is characterized by the increased expression of VEGFR2, which can modulate VEGF activity, a major mediator of angiogenesis. Src inhibitors can be used in the inhibition of VEGFR2 tyrosine kinase activity and thus decrease tumor angiogenesis. Several promising molecular targets and their associated subtypes are included in Table 1 below. The molecular pathogenesis of these subtypes is also included, specifying affected cell signaling pathways.

Both the BL1 and BL2 subtypes are characterized as basal-like according to their intrinsic BCa subtype. The BL1 subtype is further characterized by mutations in genes controlling DNA-damage response, cell division, cell proliferation, and cell cycle gene expression pathways, while the BL2 subtype is characterized by having mutations in pathways regulating glycolysis, gluconeogenesis, and growth factor signaling, in addition to those mutations found in the BL1 subtype. Due to enhanced signaling activity in pathways involved in cellular proliferation, motility, and division, the tumorigenic potential of both these subtypes is increased, which in turn facilitates tumor invasion. The M and MSL subtypes, both unclassified according to their intrinsic BCa subtype, are characterized by epithelial–mesenchymal transition (EMT), where epithelial cells are transformed into invasive, migratory mesenchymal stem cells after the loss of their cell polarity and cell–cell adhesion properties [19]. Both subtypes are also associated with dysregulations in growth factor signaling through mutations in the TP53 gene, for example, which also promotes cell proliferation. The MSL subtype is uniquely associated with mutations in genes involved in angiogenic signaling pathways, such as the VEGFC gene, which promotes angiogenesis and endothelial cell growth. The LAR subtype, classified as a luminal A intrinsic BCa subtype, is characterized by upregulations in genes involved in androgen and estrogen metabolism, such as the XBP1 and AR genes, and has dysregulated genes involved in cell proliferation. The IM subtype, characterized under the luminal B intrinsic BCa subtype, is unique in that most of the implicated signaling pathways are involved in immune signaling but are also involved in the expected cell proliferation, DNA-damage response, and cell division pathways as well. There are several genes involved in the immune signaling pathway, among which PDCD1, CTLA4, and CD274 receive particular importance due to their key roles in encoding important immune checkpoint proteins. The last subtype, UNS, is classified as a claudin-low intrinsic BCa subtype, which is involved in impaired signaling pathways for both DNA-damage response and cell proliferation and has several genes implicated in its pathogenesis.

#### 1.1.3. Current Therapies

At this time, the application of targeted therapies in TNBC treatment is limited; however, promising research in the area of personalized medicine is currently underway. Targeted therapies are specific to each molecular subtype of TNBC, as they exploit knowledge about the genomic alterations that occur in each subtype to formulate individualized treatments. Specific examples of these treatments are summarized in Table 2. For instance, the BL1 subtype is characterized by an upregulation of certain genes that are involved in cellular proliferation as well as the DNA-damage response. Thus, a potential therapeutic strategy to treat TNBCs with the BL1 subtype is to utilize antimitotic or cytostatic agents to inhibit cell proliferation, as well as to directly target products of genes implicated in pathogenesis—such as the overexpression of the PIK3CA oncogene. Mutations in this gene lead to alterations in the PI3K pathway, which regulates various cell behaviors, including proliferation, motility, and morphology [20]. Thus, a potential treatment for the BL1 subtype may be to use PI3K inhibitors to inhibit cell proliferation and migration. Following this approach with the other subtypes, BL2 subtypes may be effectively treated using growth factor receptor inhibitors, as one of the main signaling pathways implicated in BL2 pathogenesis is impaired growth factor signaling. Two such targets are the EGFR and EGF genes that are associated with the growth and progression of certain cancers. EGFR encodes the epidermal growth factor receptor, which induces signaling pathways to promote cell growth and division upon binding to ligands including the epidermal growth factor peptide, which is encoded by the EGF gene. The M subtype can be potentially treated through inhibition of gene targets that promote cell motility, including the WNT gene implicated in the Wnt/β-catenin signaling pathway. This approach can also be used for the MSL subtype, which is also characterized by the upregulation of genes involved in cell motility. MSL subtypes can also be treated with angiogenesis inhibitors, targeting vascular endothelial growth factor (VEGF) and its receptor (VEGFR), which are both involved in most cases of pathological angiogenesis seen in cancers.

The LAR and IM subtypes of TNBC are more unique in terms of their molecular signaling targets, wherein they are associated with upregulated genes involved in more complex pathways for androgen metabolism and immune signaling, respectively. For the LAR subtype, potential therapies can include androgen receptor (AR) antagonists that inhibit AR signaling, or using other nonsteroidal antiandrogens to inhibit cell growth. For the IM subtype, the most effective targeted therapies would inhibit immune checkpoint proteins, such as PD-1/PD-L1 and CTLA-4, which are usually over-expressed in cancers. Due to this, T cell activation is suppressed, resulting in tumor cell survival. Such immune checkpoint proteins are involved in suppressing immune responses, and so selectively inhibiting these molecular targets may lead to a strengthened immune response against cancer cells. Lastly, the UNS subtype has a characteristic upregulation of genes involved in DNA-damage response and cell proliferation signaling pathways, and so potential targeted therapies for this subtype can include inhibition of PLK1 and TTK amplification, both molecular targets involved in cell proliferation.

Due to various levels of pathologic overlap between the seven molecular TNBC subtypes, there is the potential that several targeted therapies can be used to treat multiple subtypes. For example, antimitotic or cytostatic agents targeting specific proteins involved in cell proliferation signaling pathways, including PI3K and Myc, can be potentially used to treat many of the subtypes. However, despite the versatility in such therapies, there are still several intrinsic disadvantages that must be considered, principally, the potential for drug resistance of tumor cells and the lack of high specificity of these therapies. With recent developments in tumor therapeutics, however, RNA-based therapies emerged. Unlike traditional therapeutics that have a more generalized action and thus lack specificity, or have a limited range of targets, RNA-based therapies have higher specificity, a wider range of targets, and good drug properties due to their ability to inhibit a variety of genes implicated in several cellular pathways that are involved in tumor cell proliferation, motility, and survival. With the ability to “target multiple key sources of multi-gene diseases such as tumors”, RNA-based therapeutics can effectively “reduce drug resistance of tumor cells and arrest the growth of advanced-stage tumors” [27]. Using antisense oligonucleotides (ASOs) is one such method that can selectively target mRNAs. Therapeutic ASOs are small molecular drugs that can modify the expression of mRNA through two key mechanisms. The first is through altering mRNA splicing, which can change the functional protein expressed, and the second is through degradation of the mRNA, by recruiting RNAase H, a “ubiquitous cellular enzyme that recognizes DNA:RNA hybrids and cleaves the RNA in the hybrid” [28]. As such, therapeutic ASOs present as a promising approach to targeted treatment of TNBC, through their ability to inhibit the translation of targeted mRNAs by preventing ribosomal binding, as well in exerting other selective effects on RNA repair, protein production, and gene expression [28].

Similar to the mode of action of ASOs, which intrinsically depend on the recognition of specific target mRNA sequences, other RNA-based therapies exist that specifically utilize different types of RNA to modulate certain effects on gene expression through silencing, or through the introduction of synthetic RNA. RNAi, for example, is an endogenous mechanism that primarily utilizes siRNA and miRNA for gene silencing effects, while therapies involving mRNA are primarily used for modifying protein expression in target cells and manipulating cellular phenotypes through the introduction of synthetic mRNA. The utility and effectiveness of these RNA-based therapies will be discussed in detail through the next sections, with special consideration to their application in the treatment of TNBC.

### 1.2. Gene Therapies Using Non-Coding RNAs

The use of non-coding RNAs (ncRNAs) presents a promising therapeutic approach for the treatment of various complex disease states, among which cancer is a prime example. ncRNAs do not encode proteins but are instead involved in several important functions, including the regulation of gene expression at both the transcriptional and post-transcriptional levels [29]. Two of the main types of ncRNAs are siRNA and miRNA. siRNAs produce a gene silencing effect by directing the degradation of specific mRNAs. In contrast, miRNAs are involved in the regulation of gene expression by blocking the translation of specific mRNAs and lead to their degradation. siRNA-based therapeutics involves the introduction of synthetic siRNA encapsulated in nanodelivery vehicles into target cells to elicit RNAi, thereby inhibiting the expression of a specific mRNA. miRNA-based therapeutics, by contrast, comprises two main approaches: miRNA inhibition and miRNA replacement. The first approach utilizes synthetic single-stranded RNA molecules antagonistically to inhibit the action of endogenous miRNAs. miRNA antagonists (or anti-miRs), as they are called, degrade endogenous miRNAs that are dysregulated and function either as oncogenes or tumor suppressors, for example, in the case of cancers [29]. The second approach employs synthetic miRNAs to mimic the function of endogenous miRNAs involved in mRNA degradation or inhibition, and gene silencing [29]. Both miRNA and siRNA-based therapeutics present significant potential in their utility in gene therapy, particularly for the treatment of aggressive diseases, such as TNBC. This section will specifically focus on elucidating the mechanisms of both siRNA and miRNA-based therapeutics, summarizing their key molecular targets and mechanisms of delivery, and discussing their efficacy in practical treatment.

### RNA-Interference Therapies

**Figure 1:**
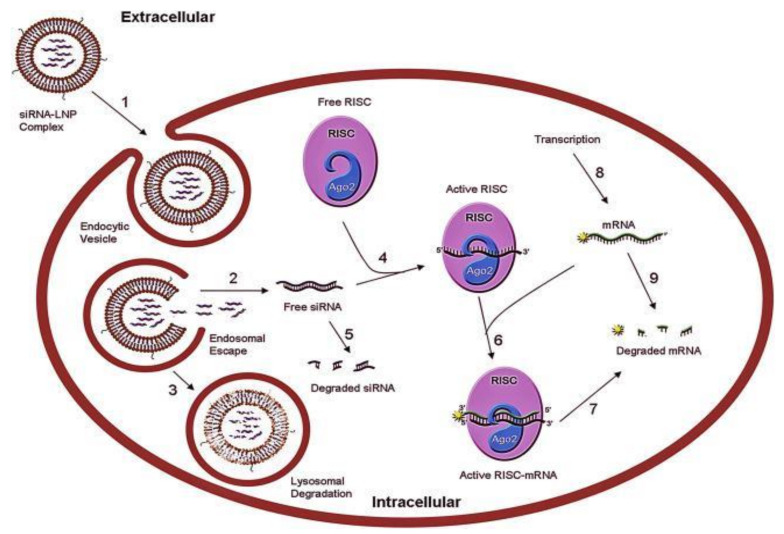
General illustration of siRNA/miRNA gene silencing mechanism: (1) siRNA-LNP complex enters target cell via endocytosis: (2) endosome is unpackaged, and siRNA escape from endosome to the cytosol, avoiding lysosomal degradation; (3) lysosomal degradation occurs; (4) free siRNA loaded onto RISC; (5) siRNA degraded in the cytoplasm; (6) active RISC formed with target mRNA; (7) target mRNA cleaved by RISC; (8) transcription rate of mRNA; (9) degradation of mRNA. LNP: lipid nanoparticle; RISC: RNA-induced silencing complex, a ribonucleoprotein complex functioning in gene silencing. Reproduced with permission from Mihaila, R. et al., Mol Ther Nucleic Acids; published by Elsevier, 2017 [32].

siRNA-based therapeutics are actively being investigated in the field of gene therapy, wherein synthetic genetic material is inserted into host target cells to treat disease. siRNA specifically silences genes that are implicated in pathogenesis and thus produces a therapeutic effect; for example, in cancer therapy, miRNAs can suppress oncogenic mRNAs from translation and thereby inhibit tumor progression (Figure 1). The first approved siRNA-based therapeutic, Onpattro^®^ (patisiran), is used to treat hereditary transthyretin amyloidosis (hATTR), a disease characterized by mutations in the gene encoding transthyretin and abnormal deposits (amyloids) of transthyretin protein, causing polyneuropathy and cardiomyopathy [30]. Patisiran specifically inhibits the hepatic synthesis of transthyretin by degrading mutant and wild-type TTR mRNA. This reduces levels of serum TTR protein, which leads to a reduction in the amyloid deposits that accumulate in different tissues [30]. Another approved siRNA-based therapeutic is Givlaari^®^ (givosiran), which is used to treat acute hepatic porphyria (AHP), an inherited metabolic disorder that leads to the accumulation of “neurotoxic precursors δ-aminolevulinic acid (ALA) and porphobilinogen (PBG)”, which can cause acute porphyria attacks. Givosiran is an aminolevulinate synthase 1 (ALAS1)-directed siRNA that prevents the accumulation of ALA and PGB by downregulating ALAS1 mRNA through hepatocyte-targeted delivery [31].

miRNA-based therapeutics has also emerged as a rapidly growing field in RNAi cancer research, though, unlike siRNA therapeutics, there have not been any approved drugs using this approach to date. Regardless, the technology has considerable potential in cancer therapeutics, due to its high versatility. Specifically, miRNA therapeutics can be used in both inhibitory and enhancing functions. Anti-miRNA therapy represents the main approach through which miRNAs are used in an inhibitory fashion, where they act as suppressors of prometastatic miRNAs. The enhancement function of miRNAs can be represented by miRNA mimicry, where synthetic miRNAs mimic the function of endogenous miRNAs involved in mRNA degradation and gene silencing. Thus, miRNA mimics can effectively enhance the function of endogenous miRNAs in gene silencing.

Miravirsen (SPC3649) is an experimental drug currently in clinical testing for the treatment of hepatitis C virus (HCV) infections and is a prime example of anti-miRNA therapy. It is a modified ASO that is complementary to mature miR-122, which is a “liver-specific miRNA that is an important host factor for the life cycle of HCV” [33]. Through inhibition of miR-122, miravirsen prevents its interaction with HCV RNA, effectively blocking HCV replication.

**Figure 2:**
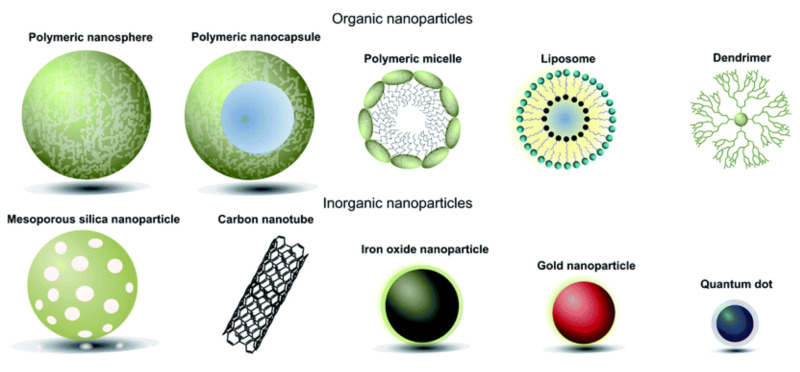
Classes of RNAi nanodelivery vehicles (subdivided into organic and inorganic nanoparticles). Reproduced with permission from Richards, D.A. et al., Chem Sci; published by Royal Society of Chemistry, 2016 [39].

Evidently, both miRNA and siRNA-based therapeutics are highly complex and targeted treatments that require specificity in their delivery. Currently, there are three principal delivery systems for RNAi therapeutics: polymer-based, lipid-based, and those using amorphous drug–polyelectrolyte nanoparticle complexes (or nanoplexes/nanocomplexes for short) [34]. Patisiran consists of synthetic siRNA encased in a lipid nanoparticle, which also contains two novel lipid excipients, a cationic lipid called DLin-MC3-DMA (or MC3), and another lipid nanoformulation called PEG2000-C-DMG (1,2-dimyristoyl-rac-glycero-3-carbonylaminoethyl-ω-methoxypolyethylene glycol-2000) [35]. Other approaches to RNAi delivery include cell-penetrating peptides, and as portrayed in Figure 2, various types of nanoparticles (NPs), including liposomes and micelles [34]. Three types of nanoparticles that are being investigated for the delivery of RNAi therapeutics are inorganic nanoparticles, polymeric nanoparticles, and lipid nanoparticles (LNPs). Inorganic NPs that have been previously used for the delivery of siRNA include silica, calcium, gold, magnesium, strontium, metal oxides, and carbon nanotubes [34]. Polymeric NPs are most commonly cationic polymer-based, where the most common cationic polymer used for siRNA delivery systems is branched polyethylenimine (PEI) [34].

Dendrimers are branched polymeric molecules that have also been used for siRNA delivery systems, where the most commonly used is poly(amidoamine) (PAMAM) [34]. They are flexible structures that can easily modify their shape and other physicochemical properties; however, there are still concerns associated with their use, including non-specific cytotoxicity, rapid clearance in vivo, and poor delivery efficiency [34]. NP delivery systems, in contrast, present higher specificity for the disease states that they are being used for. For example, the majority of in vivo delivery systems for BCa are NP-based, formed from multiple polymers mixed or conjugated to form nanoplexes (composed of polyethylene glycol (PEG) and PEI) and characterized by the loading of nucleic acids onto the surfaces of nanoplexes and utilizing layer-by-layer NPs [34]. However, LNPs specifically, such as those used for patisiran, are complex liposome-like structures that are typically composed of four main elements: a cationic ionizable lipid, a phospholipid, cholesterol, and a PEG-lipid. The cationic lipid complexes with RNA to form a core structure, which structural lipids (including phospholipids and cholesterol) envelop, and the PEG-lipid protects the NP shell [36]. LNPs have constituents similar to that of liposomes, including DOTAP (1,2-bis(oleoyloxy)-3-(trimethylammonio)propane), DOPE (dioleoylphosphatidylethanolamine), and DC-Chol (3β[N-(N′,N′-dimethylaminoethane) carbamoyl]cholesterol), but may also be composed of cholesterol analogs such as DLin-MC3-DMA, DSPC (distearoylphosphatidylcholine), DMG-PEG-2000 (1,2-dimyristoyl-rac-glycero-3-methoxypolyethylene glycol-2000), and naturally occurring phytosterols [34,36]. LNPs may also be coated with PEG, through a process called PEGylation, to confer a “stealth coating” that protects against endogenous processes including protein binding and complement activation [37]. Additionally, LNPs may be combined with both lipids and polymers to form polymer–lipid hybrid nanoparticles (PLHNPs) with lipid cores and polymeric shells, which combine the advantages of both polymeric NPs and liposomes [38].

Liposomal delivery of RNAi therapeutics involves the encapsulation of nucleic acids in small artificial vesicles that can encase both hydrophilic and lipophilic drugs. These sphere-shaped vesicles consist of a membrane composed of one or more phospholipid bilayers with an internal aqueous core, and function in intracellular delivery of encapsulated material through endocytosis. Cationic liposomes have been observed to have the highest encapsulation efficiency, attributed to their specific formulation, which confers protection to nucleic acids from degradation [34]. Cationic liposomes can be prepared from DOTAP and CD (carboxymethyl-β-cyclodextrin), DOPE, or DC-Chol [34]. The liposomal surface can also be PEGylated to further improve efficiency in delivery. This has been shown to impart several advantages, including reducing immune response, increasing half-life, and improving stability in vivo, though it may also compromise silencing efficacy by affecting cellular uptake of the liposome [34]. Some disadvantages of using liposomes in nanomedicine that must first be considered include their low solubility, high production costs, and their potential to cause hypersensitivity reactions in vivo.

Similar in structure and flexibility to liposomes, supramolecular assemblies such as micelles can also be used in RNAi-based therapeutics. Micelles encapsulate nucleic acids and drugs and are generally composed of phospholipids arranged in closed spherical monolayers, but unlike liposomes, lack an aqueous core [34]. There are three main types of micelles: normal (spherical) micelles, reverse micelles (where the orientation of the nonpolar and polar phases has been inverted), and bilayer lamellar micelles [40]. Polymeric micelles (PMs) are a subset of micelles, which are formed by the spontaneous arrangement of amphiphilic block copolymers in aqueous solutions [41]. Spherical PMs are made up of a shell of hydrophilic polymer blocks, such as polyethylene glycol or other triblock copolymers such as poly(styrene-*b*-2-vinyl pyridine-*b*-ethylene oxide) (PS-*b*-PVP-*b*-PEO), and a hydrophobic core, composed of polymers such as polypropylene glycol [42,43]. This specific architecture facilitates the loading of hydrophobic drugs into the PM core [41]. PMs can be configured in different ways, where either an inverted or normal orientation can be used in nanodelivery, and this versatility is responsible for their significant research interest [34,44]. Overall, micelles have several advantages including their relative simplicity in preparation, low toxicity, long half-life, and ability to effectively penetrate tissues; however, they can be diluted after intravenous administration [34]. Examples of micelles that can be complexed with nucleic acids include the A–B–C triblock copolymer PEG–PnBA–PDMAEMA (poly(ethylene glycol)–poly(n-butyl acrylate)–poly(2-(dimethylamino)ethyl methacrylate)), and copolymers such as PEG–PEI [34].

Cell-penetrating peptides (CPPs) are generally positively charged molecules composed of natural or synthetic short chains of linked amino acid monomers that can be used to facilitate the intracellular delivery of nanoscale particles, chemical molecules, proteins, and nucleic acids [34]. Their positive charge enables them to effectively bind to negatively charged nucleic acids, such as siRNA, to improve their stability in vivo. Three general delivery systems have been created using CPPs: CPPs modified with the polysaccharide chitosan and then used to encapsulate siRNA, CPPs loaded onto a primary delivery vehicle (such as liposomes or ultrasound-sensitive nanobubbles), and CPPs conjugated with other cationic polymers (such as PEG) or micelles to form nanocomplexes [34]. Although CPP delivery systems have shown promising results in vivo, there are two main disadvantages in their use. First, CPPs occupy a relatively new area of development, as their intracellular uptake and internalization pathways have not yet been well understood, and therefore, more research will need to define these pathways to understand the extent of application areas for CPPs. Second, CPPs appear to have relatively short blood plasma half-lives, which may affect their delivery efficacy [34].

The three NP delivery systems presented in previous sections can be used in a variety of contexts, but ultimately, the main goal for this therapeutic approach is to have specific targeting ability. RNAi can utilize these NP delivery systems to encapsulate siRNA and miRNA for targeted delivery to cells; however, specific molecular targets must first be clearly defined. As was discussed in Section 1.1.2, there are various molecular genetic targets implicated in TNBC that can be effectively targeted using RNAi, such as *TP53*. Biological features that are therapeutically targeted in various disease states are involved in the dysregulation of biological processes—in the case of patisiran to treat hATTR, for example, mutant and wild-type TTR are targeted. siRNA and miRNA can target a diverse range of molecular targets that are involved in TNBC pathogenesis, such as those implicated in dysregulated cell proliferation or motility, and growth factor signaling. Through targeted delivery of siRNA and miRNA to TNBC cells, previously dysregulated mRNAs can be inhibited from producing mutant proteins that are involved in progressing TNBC pathogenesis. Furthermore, unique characteristics of tumors can also be targeted using RNAi, such as cancer stem cells (CSCs). CSCs are located within tumors and are often implicated in several processes that are involved in cancer progression and increased aggressiveness, including metastasis, drug resistance, and disease relapse [45]. Several stem cells markers have been reported thus far, including CD24, CD44, CD133, ALDH1, and ABCG2, which can all be potential targets in RNAi therapy for TNBC [45]. Examples of specific genetic molecular targets that have been commonly targeted for TNBC can be seen summarized in Table 1, where specific targets between different subtypes of TNBC have also been delineated. In the following section, another RNA-based approach for cancer therapy will be discussed, which utilizes knowledge about a patient’s unique repertoire of tumor neoantigens expressed exclusively on malignant tissues, known as tumor-specific antigens (TSAs), to synthesize individualized neoepitope mRNA cancer vaccines that can stimulate a host immune response targeting tumors [46].

### 1.3. RNA-Immunotherapy

mRNA vaccine immunotherapy is a relatively new area of investigation within nanomedicine, which focuses on the development of personalized mRNA vaccines for the treatment of various cancers. Broadly, nucleic acid cancer vaccines contain antigens encoded by either DNA or RNA and can be further subdivided into RNA and DNA vaccines that utilize different mechanisms for therapeutic delivery. Specifically, DNA cancer vaccines consist of TSA-encoding gene(s) cloned into a bacterial plasmid [47]. The DNA plasmid is then transcribed and translated in the host, resulting in the production of the encoded antigen, which is often a protein tumor marker. These proteins are then processed into peptides and are ultimately presented on the surface of host antigen-presenting cells (APCs) in the context of major histocompatibility complex (MHC) molecules. The peptide–MHC complex is then specifically recognized by neoantigen-specific T cells, resulting in a cellular host immune response, targeted against host tumor cells bearing the specific antigens [47]. RNA vaccines, on the other hand, use “mRNA synthesized by in vitro transcription (IVT) using a bacteriophage RNA polymerase and template DNA that encodes the antigen(s) of interest” [47]. Following host administration and internalization by host cells, the mRNA transcripts are then translated, and the resulting TSAs are presented by APCs to T cells, which mount a host immune response.

Currently, there are three general approaches for the delivery of mRNA vaccine immunotherapies: naked mRNA vaccines, encapsulated mRNA vaccines, and mRNA transfected dendritic cell (DC) vaccines. Naked mRNA vaccines are characterized as injections of free mRNA formulated only in buffer and without a carrier, such as LNPs or liposomes [47]. Although numerous studies in animal models have demonstrated the efficacy of naked mRNA in inducing host immune responses, these vaccines are still limited, particularly by the “short extracellular half-life of naked mRNA due to rapid degradation by ubiquitous RNAases”, and the transiency of protein expression from naked mRNA [47]. This second limitation is particularly discouraging because it may reduce the time course of the treatment’s effect and thus may require additional clinic visits for the patient to repeat the vaccine therapy [47]. Encapsulated mRNA vaccines utilizing IVT provide a novel method for vaccine delivery. mRNA can be encapsulated in cationic liposomes, such as DOTAP, LNPs, or nanoemulsions, which improve bioavailability and physical stability, provide protection from nuclease degradation, and enhance cellular uptake and delivery efficiency [48]. For further efficiency in vaccine delivery, fully biodegradable NPs can also be engineered. Current studies have formulated such NPs to consist of a “pH responsive poly-(b-amino ester) (PBAE) core enveloped by a phospholipid shell” and have found that they can effectively deliver mRNA in vivo and elicit antitumor immune responses [47].

The immunogenicity of mRNA-based vaccines can be further increased through the addition of adjuvants. While naked mRNA vaccines “inherently possess self-adjuvanticity”, other molecules can be added to naked IVT mRNA to further enhance the vaccine’s ability to elicit an adaptive immune response, including poly I:C RNA, protamine, and CpG containing motifs [47]. The tumor microenvironment, however, is characterized by strong immune evasion and immunosuppression, which may decrease the potency of mRNA vaccines. Engineering mRNA to encode for co-stimulatory molecules, such as CD40L, CD83, CD70, and GITR, however, is a potential solution to increase the immunogenicity of mRNA vaccines to actively overcome tumor immune suppression [47].

A less-employed approach of mRNA vaccine delivery is called particle bombardment, or biolistic transfection. This method can be used to intracellularly deliver both DNA and RNA vaccines to mammalian cells through penetration of target cell membranes. This is principally achieved using IVT mRNA coated onto gold particles, which are then “accelerated toward a stopping plate by a pressurized helium pulse” [47]. Although this method has been shown to effectively deliver IVT mRNA in animal models, biolistic immunizations have yet to be translated to human clinical trials. Similarly, electroporation is another novel delivery method that can be used to introduce DNA or RNA into mammalian cells. This is by utilizing an electrical field to permeabilize cell membranes through transient pore formation, which facilitates the entry of nucleic acids and other substances (including drugs and chemicals). This delivery method can be used in the context of both RNAi and RNA immunotherapy. For immunotherapy, specifically, electroporation is used in mRNA-transfected DC vaccines, by transfecting mRNA encoding tumor antigens into DCs [49]. As professional antigen-presenting cells, DCs occupy an important role in modulating anti-tumor immunity and inducing both innate and adaptive immune responses to tumor antigens. The DC vaccination platform presents as a novel approach utilizing transfected mRNA to design highly specific anti-tumor therapies. For this approach, DCs are transfected with either tumor-associated antigen (TAA) mRNA or total tumor mRNA [47]. TAAs are specific tumor-derived molecules that are either expressed in certain cancer cell lineages or are expressed more strongly on malignant tumors, compared with healthy tissues [46].

As mentioned previously, DC transfection can be completed through electroporation, where mRNA is electrotransferred into DCs. After DCs are transfected with TAAs, they can go on to present these molecules to CD8+ T cells on MHC class I molecules. This, in turn, confers T cells with the ability to recognize these specific antigens on tumor cells and enables them to stimulate active immune responses targeting tissues expressing those specific antigens. In this way, DCs can be used to effectively target tumors in an antigen-specific manner. Similarly, total tumor mRNA can also be used to elicit host immune responses against tumors utilizing a specific profile of derived antigens. This method utilizes cancer-specific RNA, thus eliminating the need for the identification of antigens expressed by the patient’s tumor [47]. With the entire spectrum of TSAs displayed, the immune system can more efficiently stimulate immune responses against tumors by utilizing only effective antigens, which also reduces the risk of escape from mutants [47].

**Figure 3:**
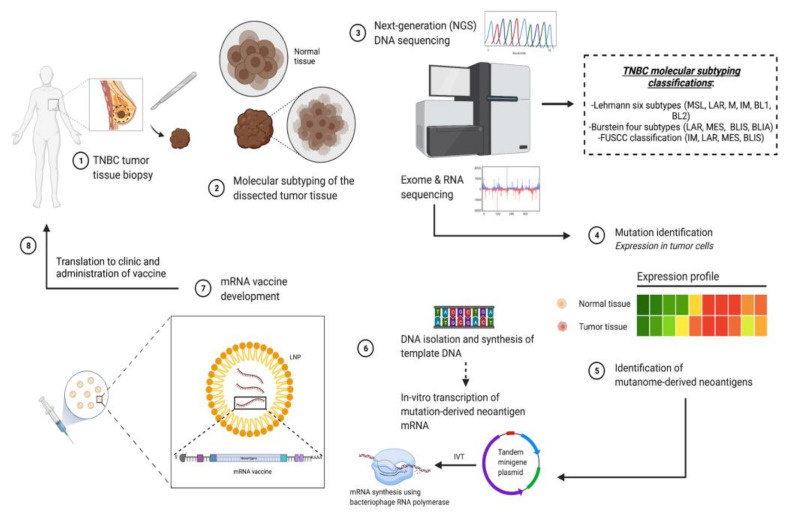
Development of personalized mRNA vaccines for TNBC.

Molecular targets using RNA-based immunotherapies are characteristically different from those discussed for RNAi, where instead of targeting dysregulated proteins, antigenic substances present on tumors are instead targeted to elicit host immune responses [50]. Immunotherapeutic targets in TNBC are generally characterized as TAAs, and among the most prominently over-expressed antigens on TNBC tumors are cancer-testis (CT) antigens, which are preferentially expressed as a result of epigenetic changes [51]. There have been over 150 CT antigens documented thus far, among which MAGE-A, NY-ESO-1, FOXM1, ATAD2, and SPANXB1 present as common features of TNBC [51,52,53,54]. Other TAAs commonly associated with TNBC include mesothelin (MSLN), mucin 1 (MUC1), folate receptor alpha (FOLR1), and trophoblast cell surface antigen 2 (Trop2) [53,55]. Immunotherapeutic strategies that can be used to target TAAs can include the use of naked monoclonal antibodies (mAbs), immunoconjugates (including immunotoxins and drug-conjugated mAbs), oncolytic virotherapy, CAR-T cell therapies, or mRNA cancer vaccines, including those approaches previously mentioned above. Figure 3 below describes the application of TAAs in the development of mRNA vaccines for TNBC. Initially, TAAs are first identified in resected tumor tissue through molecular subtyping and sequencing. After this, mutanome-derived neoantigens are identified to begin mRNA vaccine development, which eventually leads to clinical translation of the vaccine and administration to the patient.

Combination immunotherapies can also be implemented for TAA-targeted therapies, wherein two individual therapies are used in conjunction for treatment. An example is the use of TAA-targeted mRNA nano-vaccines with CTLA-4 inhibitors [55]. For this treatment strategy, tumors are targeted by the host immune system, along with the immune checkpoint receptor CTLA-4 being blocked and deactivated (i.e., CTLA-4 blockade) [55].

**Figure 4:**
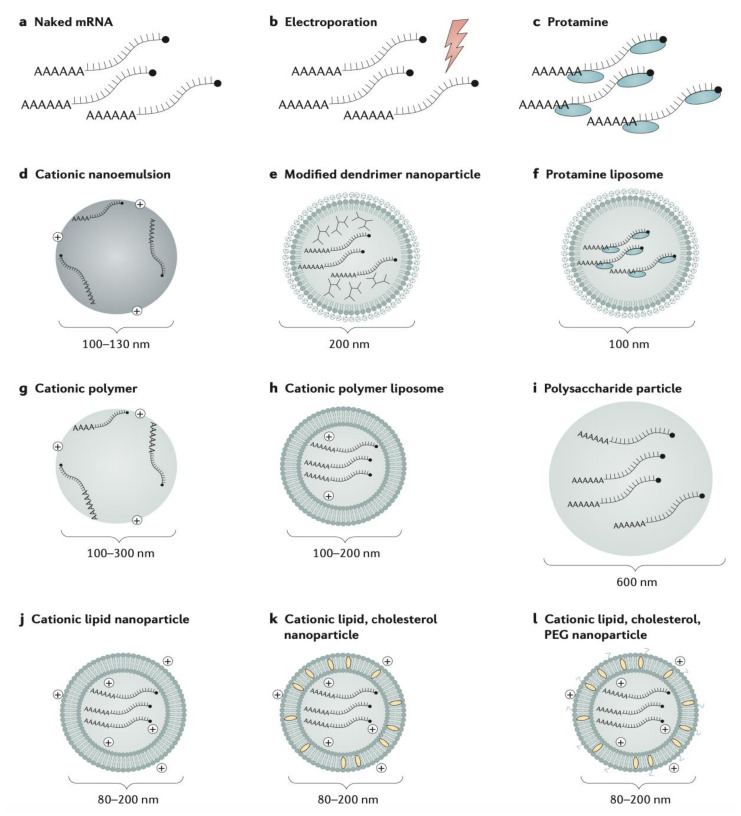
mRNA vaccine delivery vehicles. Reproduced with permission from Pardi, N. et al., Nat Rev Drug Discov; published by Springer Nature, 2018 [57].

Delivery of these immunotherapies will be varied but would generally consist of the subcutaneous or intravenous infusion of therapeutic mAbs, CAR-T cells and mRNA transfected DCs, and the NP, liposomal, or nanoemulsion-mediated delivery of mRNA cancer vaccines (Figure 4). Overall, as a new and emerging therapeutic strategy for cancer treatment, RNA-immunotherapy possess significant potential in its clinical applications. Alongside RNA-based interference therapies, RNA-based immunotherapy can be effectively used to treat various disease states, including aggressive cancers, such as TNBC. While the applications of these novel technologies are certainly promising, there are understandably still preclinical safety challenges that have yet to be addressed before translating them into clinical practice. For one, not many studies have been conducted on evaluating the toxicity profiles of these therapeutics or describing their pharmacokinetic and pharmacodynamic profiles, and so this is a key area of research for the future. While standard toxicity studies for RNAi have been conducted in the past, many of the applications that were discussed have not been studied yet. Thus, this demonstrates the need for more preclinical work in this area before introducing these novel technologies and ushering in the next generation of cancer therapeutics [56].

### 1.4. Combination Therapies Using Chemotherapeutics and RNA-Based Therapies

RNA-based therapies can be used in combination with traditional chemotherapeutics to enhance therapeutic efficacy and improve patient responsiveness to treatment through synergistic therapeutic effects. Several chemotherapeutics that have been used in combinatorial cancer therapy include benzethonium chloride (BZN), paclitaxel, cabazitaxel, doxorubicin, and orlistat. BZN has been used in nanocomplex formulations with Bcl-2-targeting siRNA and has demonstrated therapeutic efficacy in vivo [58]. Hybrid NPs have also been designed to co-deliver paclitaxel and anti-miRs (specifically for miR-221/222) to TNBC cells. miR-221/222 are oncogenic miRNAs over-expressed in TNBC and are involved in various prosurvival functions, including cancer initiation and progression, EMT, and resistance to certain chemotherapeutics [59]. Combination therapy using anti-miRs demonstrated the increased therapeutic efficacy of paclitaxel through inhibition of the proliferative mechanisms of miR-221/222 [59]. miRNA inhibition was also used in the hydrophilic NP co-delivery of anti-miRs, specifically for miR-21, and orlistat, an anti-obesity agent [60]. miR-21 is an endogenous miRNA upregulated in TNBC, that has various oncogenic functions including antiapoptotic activity, tumor proliferation, and drug resistance [60]. Combination treatment was shown to have significant effects in apoptotic induction, demonstrating promising efficacy and future use in TNBC therapy [60].

Co-delivery of cabazitaxel and siRNA targeting IKBKE, an oncogene present in TNBC, using hybrid nanocomplexes also demonstrated improved therapeutic efficacy, wherein cabazitaxel enhanced the activity of the IKBKE siRNA [61]. Exosome-mediated co-delivery of doxorubicin, and miR-159 also demonstrated significant results in TNBC therapy. This approach involved miRNA replacement using miR-159, an endogenous miRNA inversely correlated with BCa incidence and progression, and which demonstrated synergistic efficacy through improved anticancer effects and reduction of adverse effects [62]. Other studies utilizing this approach included the NP co-delivery of doxorubicin, separately with miR-34a and miR-542-3p, both of which are endogenous tumor suppressor molecules [63,64].

**Figure 5:**
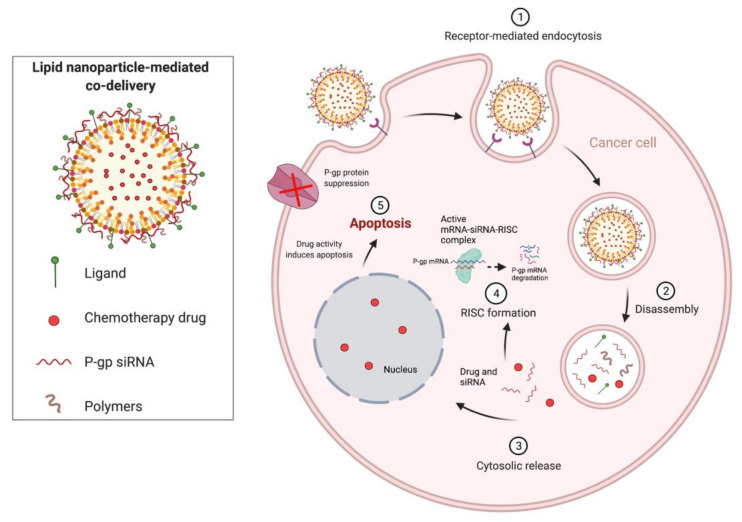
Combination cancer therapies in overcoming MDR.

Combination cancer therapies can be used to improve the efficacy and safety of current treatments utilizing small-molecule anticancer drugs, as well as amplifying the effects of RNAi. Further, combinatorial therapeutic approaches can also be used to overcome multidrug resistance (MDR), a considerable challenge in cancer treatment. Co-delivery of RNAi and anticancer drugs can produce a synergistic effect that can lead to improved treatment efficacy. As shown in Figure 5 below, siRNA targeting P-glycoprotein (P-gp), an efflux protein that mediates MDR, in combination with an anticancer agent can lead to therapeutic synergism, where P-gp siRNA inhibits expression of P-gp, and the anticancer agent causes apoptosis.

**Figure 6:**
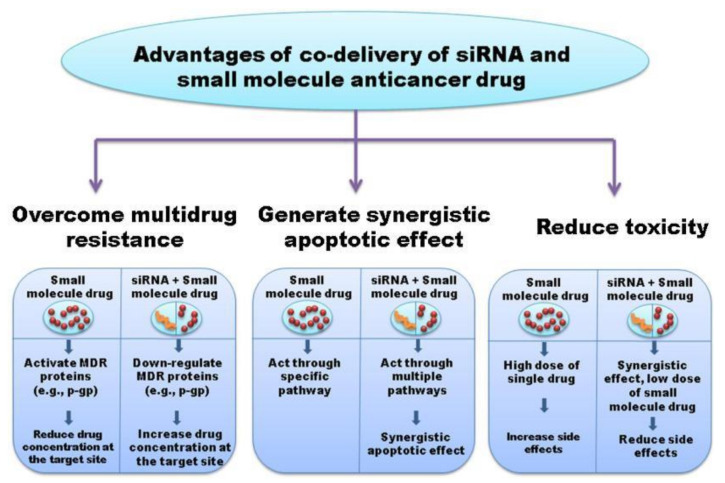
Advantages of combinatorial therapy with RNAi and anticancer agents. Reproduced with permission from Saraswathy, M. et al., Materials Today; published by Elsevier, 2014 [66].

With downregulation of P-gp, the concentration of the anticancer agent can be effectively increased at the target site, thus leading to greater therapeutic efficacy [65]. As such, RNAi can be used to effectively alter oncogenic features in TNBC cells, for example, that make them more conducive to apoptosis. Combinatorial therapies are also advantageous because they can generate synergistic apoptotic effects that are mediated through multiple pathways, which can also help prevent chemoresistance, as illustrated in Figure 6. Furthermore, combination treatments can also reduce the toxicity associated with cancer monotherapy, as high doses are often required to achieve a therapeutic response when using anticancer drugs alone. However, with the combined use of RNAi and anticancer drugs, the synergistic therapeutic effect decreases the amount of small molecule drug required, and thus also reduces the side effects from the drug. While there are still associated disadvantages with the use of combination therapies, including the potential risk for the development of novel adverse reactions, the ultimate utility of this approach is promising with significant clinical impact when optimized strategies are established. The delivery of these combination therapies, however, still presents a significant problem requiring additional research. As was described previously, several co-delivery systems have already been designed for combinatorial therapeutics, including nanocomplexes, exosomes, polymer hybrid NPs, and hydrophilic NPs, composed of formulations including hyaluronic acid and chitosan.

**Figure 7:**
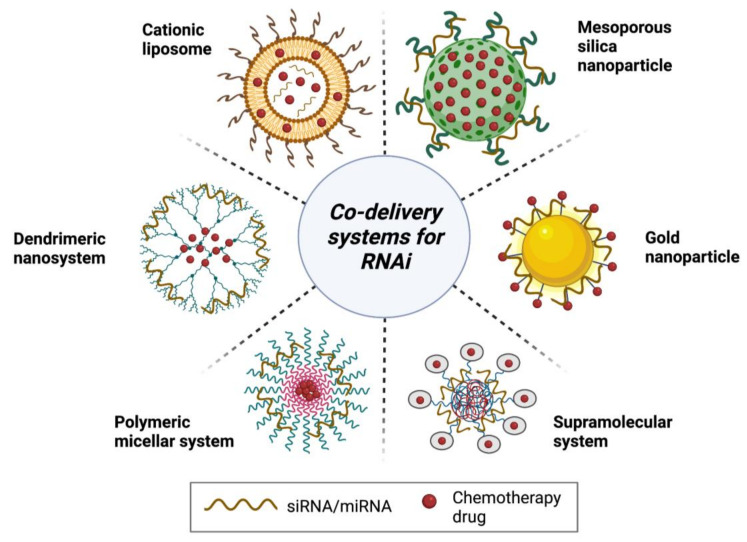
Selected co-delivery systems for combinatorial therapeutics [67]. Reproduced with permission from Wang, M. et al., Colloids Surf B Biointerfaces; published by Elsevier, 2017 [68].

As illustrated in Figure 7, co-delivery systems are formulated similarly to therapeutic delivery vehicles used by RNAi, where dendrimers, micelles, inorganic NPs, and supramolecular assemblies such as liposomes and other bioengineered systems are also utilized to deliver RNAi and anticancer drugs. Importantly, the composition of these co-delivery systems depends on various physicochemical characteristics that must be individually considered. For example, Figure 7 shows the difference in the specific orientation of the chemotherapy drug to the siRNA. This characteristic is unique to each delivery system and is dependent on several factors including the hydrophilicity of the drug, electrostatic interactions between the charged RNA, and the surface chemistry and chemical stability of the delivery vehicle, including its surface functionality and charge. Consideration of all these factors is an important step to ensuring the successful development of a delivery system that enhances the therapeutic effect of the combination therapy, by increasing its bioavailability, chemical stability, and specificity, among other qualities.

Overall, combinatorial therapeutics presents a promising avenue for future TNBC research, as well as for other diseases. The increased efficacy of combining treatment approaches, as well as the improved quality-of-life outcomes from reduced drug toxicity, are both considerable advantages that can be gained from this modality. As discussed earlier, RNA-based interference therapies also present as a promising treatment approach in the future; however, because combinatorial therapeutics enhance the therapeutic effects of such technologies, increased research efforts must be focused on optimizing this approach for use in the clinic. The next section describes specific clinical applications of RNA-interference and combinatorial therapies for the treatment of TNBC and also delves into the ongoing challenges with the use of RNAi in TNBC molecular therapy.

## 2. Therapeutic Applications of RNA-Based Methods for Treatment of TNBC

### 2.1. Pre-Clinical Progress of RNA-Based Therapies

**Figure 8:**
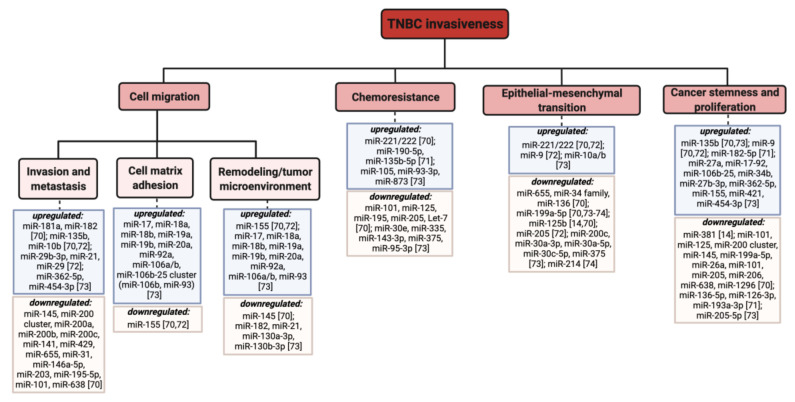
Diversity of select miRNAs contributing to TNBC invasiveness [14,69,70,71,72,73]. Adapted from [74].

Preclinical development of RNA-based therapies has been ongoing for several years as novel approaches have been routinely developed and periodically introduced. Several promising preclinical studies are ongoing for TNBC treatment, many of which involve RNA-based therapies. To date, there have been several innovative therapies that have been introduced, including using novel small molecule chemotherapeutics, various immunotherapies such as those discussed earlier in Section 1.3, ASOs, and nanodelivery of both small molecule drugs and RNAi. However, despite being a relatively new approach to TNBC treatment, RNAi has been a prominent area of research in recent years. However, interestingly, RNAi using miRNA has seen more progress than siRNA in preclinical investigation. This may be because miRNA is slightly more versatile in terms of its utility, as it can be used in both inhibitory and enhancing functions, where anti-miRs can be used to target oncogenic miRNAs, and synthetic miRNAs can be used to mimic the function of endogenous oncosuppressor miRNAs. Figure 8 below shows a collection of miRNAs that are generally seen to contribute to TNBC invasiveness, where oncogenic miRNAs (oncomiRs) are upregulated and oncosuppressor miRNAs are downregulated. As discussed, miRNAs are involved in various cellular functions, and dysregulation of miRNA expression contributes to an imbalance, which either suppresses or promotes these cellular functions. Various preclinical studies have been involved in studying the role of miRNAs in regulating these functions and have been investigating ways to either inhibit or promote their expression to control tumor invasiveness.

The table below shows some of the current miRNAs that have been investigated in preclinical studies as potential TNBC therapies, other than those already included in Figure 8. Included in the table are also a few specific molecular targets commonly seen in TNBC that are also being investigated for use with both siRNA and shRNA. shRNAs are artificial RNA molecules that are another form of RNAi, used to silence target gene expression, and are precursors of siRNAs, synthesized within the cell by DNA vector-mediated production [75]. They also use a similar cellular mechanism (RISC) to siRNA and miRNA molecules for gene silencing (see Figure 1) [75]. As was described previously in Table 1, there are various molecular genetic targets in TNBC and many are associated with specific subtypes. The molecular targets described in Table 3 present a promising avenue of research in RNA-based therapies using both siRNAs and miRNAs. While siRNA is a comparatively more well-understood field in RNAi, miRNA is also an important approach that is unique in that it can be utilized in both mimetic and antagonistic functions.

**Figure 9:**
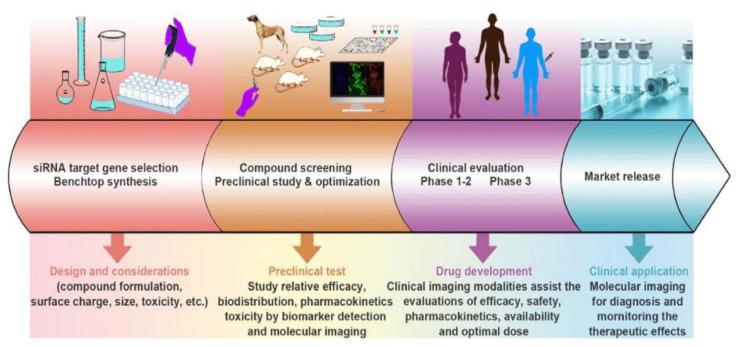
Workflow process of developing RNA-based therapies for clinical applications. Reproduced with permission from Wang, J. et al., Adv Drug Deliv Rev; published by Elsevier, 2016 [103].

There has been significant progress in developing RNA-based therapies for a wide variety of diseases, although primarily preclinically for TNBC to date. Although there are several promising early-stage studies for TNBC using RNAi and RNA immunotherapy as was described in Section 1.3, and combinatorial RNA-based therapies, described in Section 1.4, it is important to recognize that there is still a significant amount of work until these therapeutic agents/strategies will advance to clinical evaluation. As shown in Figure 9, preclinical studies are currently the second of the four-step workflow process of developing RNAi (and other RNA-based) therapeutics and translating them for clinical applications. While there is still considerable time before these studies are advanced to the patients, the significant progress that has been made recently is certainly promising and serves to demonstrate the rapid developments that have contributed to TNBC treatment thus far.

### 2.2. Current Clinical Trials Using RNA-Based Therapies

RNA-based therapies have been increasing in recent years to treat a variety of conditions, among which TNBC has been an active area of research. To date, there have been limited clinical studies that have investigated RNA-based therapeutics for TNBC, three of which are described below in Table 4. A fourth study utilizing therapeutic ASOs was also included to demonstrate the diversity of studies that have been introduced for the treatment of TNBC. Specifically, this study utilized the ASO named AZD8701 to selectively target FOXP3 mRNA for degradation and inhibit cancer immunosuppression [104]. The study compared the efficacy of AZD8701 monotherapy versus combination therapy with the immune checkpoint inhibitor durvalumab. Durvalumab is a fully human monoclonal antibody that blocks PD-L1 from binding to its receptors, PD-1 and CD80, and thus inhibits cancer immunosuppression and enhances T cell antitumor activity [105]. The first study listed investigates mRNA-2752 monotherapy versus combination therapy, also with durvalumab. mRNA-2752 is an LNP encapsulating mRNAs that encode human OX40L, a T cell co-stimulator, and the pro-inflammatory cytokines, IL-23 and IL-36γ [106].

The second study, Mutanome Engineered RNA Immunotherapy (MERIT), is an mRNA cancer vaccine trial that utilizes individualized cancer immunotherapy (IVAC), where immunogenic RNA vaccines are designed specifically for a given patient’s tumor antigen-expression profile. Two complementary strategies are used in the trial, namely the WAREHOUSE and the IVAC^®^ MUTANOME concepts, which refer to the approaches used to identify suitable molecular targets for RNA-based immunotherapy. The first concept essentially uses a patient-specific liposome complexed with mRNA of pre-identified immunogenic TAAs commonly expressed in TNBC. The second concept utilizes a two-step process, in which first, tumor-specific mutations are identified by next-generation sequencing, and second, in which on-demand RNA manufacturing is used to target neo-antigens derived from the established mutated epitopes in individual patients. In this way, RNAs are synthesized de novo to target individual tumor mutations. These two IVAC approaches can also be combined to target the entire range of antigens selectively expressed on tumors, acknowledging the heterogeneity of tumors between patients. Lastly, the third study is a current trial investigating CAR-T therapy for TNBC. Specifically, it focuses on the safety and efficacy of intratumorally administering autologous T cells in patients, redirected to target the c-Met tyrosine kinase receptor that is over-expressed in TNBC.

All the studies mentioned above present novel RNA-based therapies for the treatment of TNBC. However, upon a review of current clinical trials for TNBC treatment, it was found that most of the studies are using traditional small molecule chemotherapeutics and modern chemotherapy. Other emerging studies are investigating the efficacy of genomically directed therapies for TNBC, in turn providing personalized therapies by sequencing patient tumor total RNA or determining miRNA profiles in patients with TNBC. As such, it is evident that RNA-based therapies still comprise a relatively new and developing field that is slowly increasing in momentum for TNBC treatments. Interestingly, most of the studies that were identified in Table 4 are either still active or are currently recruiting, indicating that RNA-based therapies are gaining headway among clinical studies. However, it is important to recognize that such therapies are still undergoing rapid developments and thus will require more time to progress through bench-to-bedside translation before making their way to TNBC patients.

## 3. Future Perspectives and Conclusions

### 3.1. Ongoing Challenges with siRNA, miRNA, and mRNA Therapies

RNA-based therapies occupy a highly diverse and versatile field that can be leveraged as advanced treatments for TNBC. However, despite the significant progress described in previous sections, there remain several challenges that must be overcome before this therapeutic approach can be translated to the clinic. In Table 5, some of the advantages and disadvantages of three of the major forms of RNA-based therapies—RNAi, vaccine immunotherapy, and combination therapy—are listed. Specifically, one of the main challenges facing RNAi is the lack of specificity of miRNA, as due to imperfect binding to target mRNA, a single miRNA may target and degrade many sets of similar target mRNAs [34,107]. Another prominent concern is the nuclease-mediated degradation of naked siRNAs and miRNAs that can occur when these molecules are injected systemically. In addition to this, naked siRNAs and miRNAs can experience repulsion at the cell membrane level due to their negative charge at normal pH, generally have poor tissue penetration, and can cause non-specific immune stimulation. Although some of these challenges can be mitigated using various delivery vehicles, such as liposomes and cationic lipids, these vehicles also have associated disadvantages including systemic toxicity. Ideally, the delivery vehicles used for RNAi should satisfy several requirements to ensure maximum efficacy, including protecting siRNA and miRNA from degradation during systemic circulation, internalization in cells (endosomal trap), accumulating in the target tissue, withstanding prolonged circulation without being cleared by renal filtration, and have favorable characteristics, including biocompatibility, biodegradability, non-toxicity, and non-immunogenicity [34,108].

DC vaccines are also a novel RNA-based therapeutic that can be implemented for use in TNBC treatment. As was discussed in Section 1.3, the biologic activity of DC vaccines stems from electroporated mRNA derived from TAAs, which promote immunogenicity. The clinical administration of DC vaccines is first achieved by using leukapheresis to harvest monocytes from peripheral blood. Subsequently, immature and mature DCs are generated through culturing with IL-4 and GM-CSF and are then exposed to activating factors for maturation [109]. Next, a tumor biopsy is taken from the patient, after which TAA mRNA or total tumor mRNA is electroporated into mature DCs. The resulting antigen-loaded DCs are then cryopreserved and injected back into the patient [109]. In this process, it is evident that DC vaccines are a considerably expensive and difficult treatment modality to produce, which is one of its main drawbacks. In addition, although this approach produces a highly immunogenic cell-based vaccine, there may be several adverse effects associated with its administration, and so these considerations must be carefully examined before fully translating this therapy to the clinic.

Finally, combinatorial therapeutics, utilizing both RNAi and traditional chemotherapeutics, remain an active area of investigation. This field was developed in response to the various limitations that exist with RNAi monotherapy, including their restriction to mainly indirect actions involved in gene regulation, concerns about bioavailability, and several others discussed above. Combination therapies effectively address these limitations by improving the therapeutic potential of RNAi, as well as mitigating many of the disadvantages that exist with current chemotherapeutic small-molecule drugs. For example, using RNAi along with small-molecule drugs can help overcome MDR by downregulating MDR proteins, such as ABC transporters. Secondly, using both therapeutics enables for a stronger therapeutic effect due to synergism, and thirdly, many of the toxicities and associated side effects that occur with the use of chemotherapeutics can be prevented due to a lower dose of the small-molecule drug being required. However, using combination therapies also has associated disadvantages, including the potential for novel adverse reactions to develop with concurrent use of the two disparate therapeutic approaches. Furthermore, combination therapies are also considerably costly to implement, and so their long-term application may not be feasible. Ultimately, with all the therapeutic approaches described, the costs of the therapies must be weighed with their benefits, and if it is seen that their benefits far outweigh their costs, then it would be prudent to translate those therapies into the clinic. In the case of combination therapies, because there are numerous benefits associated with their use, such therapies will likely be seen in the clinic more readily. This is the same for the other treatment modalities discussed, which though are lacking in certain areas, with additional research and modifications, have the potential to be implemented in the clinic alongside combination therapies for future applications in TNBC treatment.

### 3.2. Future Use in TNBC Molecular Therapy

This review discussed various targeted therapies available for TNBC, among which RNAi, immunotherapy, and combination therapies were present as the promising approaches. All these approaches have the potential to be implemented in TNBC molecular therapy and translated clinically, though understandably, this will take time. Certainly, in targeting various unique pathological distinctions in TNBC, these RNA-based poly- and monotherapies present novel approaches for treating TNBC and are promising for future use. The notable success of mRNA vaccines in the management of the COVID-19 pandemic speaks both to the efficacy and the considerable adaptability of RNA-based therapies, which will likely experience a strong resurgence in the coming years for future cancer therapies [121,122]. Although there are certainly areas where modifications still need to be made to improve these therapeutic approaches and further substantiate their efficacies, these therapies certainly hold great potential for treating aggressive cancers such as TNBC.

In summary, targeted RNA-based molecular therapy of TNBC may be effectively achieved through various modalities, including RNAi, combination therapy, and vaccine immunotherapy. Of those discussed, immunotherapies and combination therapies represent innovative therapeutic approaches with great potential given their substantial recent advances. However, based on current clinical trials, combination therapies, compared to stand-alone immunotherapies, will likely be incorporated into TNBC treatment paradigms sooner in the near-term. Alternatively, non-RNA-based treatments, such as immunochemotherapy or radioimmunotherapy, are also both potential avenues of research that have not yet been studied as extensively for TNBC treatment. Ultimately, however, through additional research efforts devoted to addressing their key limitations, all the RNA-based therapies reviewed possess significant potential for future use in TNBC molecular therapy.

## Figures and Tables

**Table 1:** Molecular classification of TNBC subtypes according to genetic abnormalities and implicated signaling pathways [7,10,11,12,14,15,16,17,18].

TNBC Molecular Subtype	Intrinsic BCa Subtype	Signaling Pathway(s)	Molecular Genetic Target(s)
BL1 (basal-like 1)	Basal-like	DNA-damage response	*RAD51*, *CHEK1* [10]; *ATR* [11]; *BRCA2* [11,12]; *CTNND1*, *TOP2B*, *CAMK1G* [12]; *TP53* [12,14]
		Cell division	*NRAS* (N-Ras) [11]; *RB1* [12,14]
		Cell proliferation	*PARP1*, *PLK1*, *TTK*, *AURKA/B* [10]; *MKI67* (Ki-67) [11]; *BRCA1* [11,12]; *MAPK13*, *SMAD4*, *STAT4*, *CDKN2A*, *PIK3CA* (PI3K), *MDC1*, *PPAR1* [12]; *MYC* [11,12,14]; *PTEN*, *TP53*, *RB1* [12,14]; *CDCA4*, *RHOA*, *AKT1* [14]
		Cell cycle gene expression	*BRCA1* [11,12]; *KDM6A* (UTX), *STAT4* [12]; *CDCA4* [14]
BL2 (basal-like 2)	Basal-like	DNA-damage response	*MTOR* [10]; *TP53* [11,12]
		Cell division	*EGFR* [11]; *RB1* [12]; *PTEN* [12,17]
		Cell proliferation	*TP63* [10]; *EGFR* [11]; *MET* (c-Met), *EPHA2* [10,11]; *TP53* [11,12]; *RB1*, *IGF1R*, *MYC*, *BRCA1* [12]; *AKT1*, *TGFB1*, *E2F2*, *RHOA*, *CXCL8* [14]; *PIK3CA* (PI3K), *PIK3R1* [17]
		Glycolysis, gluconeogenesis	*CDKN2A*, *KDM6A* (UTX) [12]; *TP53* [11,12]
		Growth factor signaling	*EGF/EGFR* [10]; *BRCA1* [12]; *TGFB1* [14]
M (mesenchymal-like)	Unclassified/luminal B	Cell motility	*PIK3CA* (PI3K) [10,12]; *WNT* [11]; *PTEN*, *RB1*, *TP53* [12,14]; *PDGFA/B*, *KIT* [15]
		DNA-damage response	*MTOR* [10]
		Cell differentiation	*FGFR1* [10]; *PIK3CA* (PI3K) [10,12]; *ALK*, *TGFB1* (TGF-β1) [11]; *KIT, CTNNB1* (β-catenin), *SFRP4*, *TCF4* [15]
		Epithelial–mesenchymal transition	*TP53* [12,14]; *MMP2*, *TWIST*, *SNAI2*, *TCF4* [15]
		Growth factor signaling	*PDGFRA* (PDGFRα) [10]; *TGFB1* (TGF-β1) [11]; *TP53* [12,14]; *PDGFA/B*, *IGF1* [15]
		Cell proliferation	*IGF1R*, *SRC*, *PDGFRA/B* [10]; *WNT*, *ALK*, *TGFB1* (TGF-β1) [11]; *MYC* [12]; *PIK3CA* (PI3K) [10,12]; *RB1* [12,14]; *SNAI2*, *PDGFA/B*, *IGF1*, *KIT*, *CTNNB1* (β-catenin), *DKK2/3*, *SFRP4*, *TCF7L2*, *FZD4* [15]; *LGR6* [18]
MSL (mesenchymal stem-like)	Unclassified	Cell motility and adhesion	*TGFBR3* [10]; *RB1*, *TP53*, *VCAM1* [12]; *PIK3CA* (PI3K) [10,12]; *CCL2* [14]; *WNT*, *KIT*, *PDGFA/B* [15]
		Cell differentiation	*MAP2K1*, *FGFR1* [10]; *MAPK1/3* [11]; *PIK3CA* (PI3K) [10,12]; *KIT*, *CTNNB1* (β-catenin), *SFRP4*, *TCF4* [15]
		Cell proliferation/stemness	*SRC*, *MAP2K1*, *NFKB1*, *IGF1R* [10]; *EGFR* [11]; *TP53*, *NF1*, *RB1*, *ABCA8*, *PROCR*, *ENG*, *PER1*, *ABCB1*, *TERT2IP*, *BCL2*, *BMP2*, *THY*, *HOXA5*, *HOXA10*, *MEIS1*, *MEIS2*, *MEOX1*, *MEOX2*, *MSX1*, *THY1*, *BRCA1* [12]; *PDGFRA/B* [10,11,12]; *PIK3CA (PI3K)* [10,12]; *SNAI2*, *WNT*, *PDGFA/B*, *IGF1*, *KIT*, *CTNNB1* (β-catenin), *DKK2/3*, *SFRP4*, *TCF7L2*, *FZD4* [15]; *ALDHA1* [12,15]
		DNA-damage response	*MTOR* [10]
		Epithelial–mesenchymal transition	*TP53* [12]; *MMP2*, *TWIST*, *SNAI2*, *TCF4* [15]
		Angiogenesis	*PDGFRA/B* [10,11,12]; *PIK3CA* (PI3K) [10,12]; *TP53*, *HRAS, BRAF*, *CDKN2A*, *KRAS*, *NF2*, *ENG*, *ITGAV*, *NT5E* [12]; *CCL2* [14]; *PDGFA/B* [15]; *VEGFC* [16]
		Growth factor signaling	*MAP2K2* [10]; *TP53*, *BMP2*, *NGFR*, *BRCA1* [12]; *KDR* (VEGFR2) [11,12]; *EGFR* [11,14]; *PDGFA/B*, *IGF1* [15]
LAR (luminal androgen receptor)	Luminal A	Androgen, estrogen metabolism, porphyrin, and lipid metabolism	*AR* [10,11]; *FOXA1*, *XBP1*, *KRT18* [11]; *APOD*, *CDH1* [12]; *PIK3CA* (PI3K) [10,12]
		Steroid synthesis/regulation	*PTEN*, *DHCR24*, FASN, *FKBP5* [12]
		Cell proliferation	*HSP90AB1*, *FGFR4* [10]; *ALCAM*, PIP, *SPDEF*, *CLDN8*, *RB1*, *MYC* [12]; *PIK3CA* (PI3K) [10,12]; *AKT1* [14]; *CCND1* [15]
		Molecular apocrine subtype	*PIK3CA* (PI3K) [10,12]; *RB1*, *TP53*, *PTEN* [12]
IM (immunomodulatory)	Luminal B	Immune signaling	*BTK* [10]; *JAK1/2*, *STAT1/4*, *IRF1/7/8* [10,11]; *TNF* [11]; *TP53*, *CTNNA1*, *DDX18*, *HUWE1*, *NFKBIA*, *APC*, *BRAF*, *MAP2K4*, *RB1* [12]; *PDCD1* (PD-1), *CTLA4*, *CD274* (PD-L1), *IFNG*, *IFNA1*, *PTEN* [14]
		Cell proliferation	*NFKB1* [10]; *TNF* [11]; *TP53*, *HUWE1*, *APC*, *MAP2K4*, *RB1*, *MYC* [12]; *AKT1*, *RHOA* [14]
		Cell differentiation	*CTNNA1*, *HUWE1*, *BRAF*, *MAP2K4* [12]
		Cell division	*DDX18*, *APC*, *BRAF*, *RB1* [12]
		DNA-damage response, growth factor signaling	*LYN* [10]; *TP53* [12]
UNS (unstable)	Claudin-low	DNA-damage response	*RAD51*, *CHEK1*, *PARP1* [10]
		Cell proliferation	*PARP1*, *PLK1*, *TTK*, *AURKA/B* [10]

**Table 2:** Potential targeted drug therapies for the treatment of specific TNBC molecular subtypes [10,12,14,21,22,23,24,25,26].

**TNBC Molecular Subtype**	**Therapeutic Strategies**	**Targeted Therapies**
BL1 (basal-like 1)	-Inhibit cell proliferation and DNA damage-response	-Antimitotic agents -Cytostatic agents -PARP inhibitors -DNA synthesis inhibitors -PI3K inhibitors -MYC inhibitors -Aurora kinase inhibitors
BL2 (basal-like 2)	-Inhibit growth factor signaling (EGF/EGFR) -Inhibit cell division and proliferation	-Antimitotic agents -Cytostatic agents -PARP inhibitors -Growth factor receptor inhibitors -mTOR inhibitors -PI3K/Akt inhibitors -IGF1R inhibitors -RAS/MAPK inhibitors
M (mesenchymal-like)	-Inhibit cell migration -Inhibit Wnt/β-catenin signaling pathway	-Tyrosine kinase inhibitors (TKIs) -mTOR inhibitors -Growth factor receptor inhibitors -PI3K inhibitors -Src inhibitors -Eribulin mesylate -Wnt/β-catenin inhibitors
MSL (mesenchymal stem-like)	-Inhibit cell migration -Inhibit PDGFRα/β -Inhibit EGF/EGFR/VEGFR2	-Src inhibitors -Growth factor receptor inhibitors -mTOR inhibitors -PI3K inhibitors -MAPK inhibitors -Wnt/β-catenin inhibitors -Angiogenesis inhibitors (antiangiogenics)
LAR (luminal androgen receptor)	-Inhibit AR signaling -Inhibit *FOXA1* and targets involved in cell differentiation (*ERBB4*)	-PI3K inhibitors -AR antagonists (steroidal antiandrogens) -Nonsteroidal antiandrogens -mTOR inhibitors -Hsp90 inhibitors
IM (immunomodulatory)	-Inhibit immune checkpoint proteins including PD-1/PD-L1 and CTLA-4	-MEK inhibitors -Cytostatic agents -PARP inhibitors -Immune checkpoint inhibitors
UNS (unstable)	-Inhibit PLK1/TTK amplification	-PLK1 inhibitors -TTK inhibitors
**Select Examples of Current Chemotherapy Drugs**		
Antimitotic agents: platinum salts—cisplatin (Platinol^®^), taxanes—paclitaxel (Taxol^®^), docetaxel (Taxotere^®^), cabazitaxel (Jevtana^®^), vinca alkaloids—vincristine (Oncovin^®^), vinblastine (Velban^®^), vinorelbine (Navelbine^®^) ○ used for testicular, ovarian, bladder, prostate, lung, and other cancers PARP inhibitors: olaparib (Lynparza^®^), rucaparib (Rubraca^®^), niraparib (Zejula^®^), talazoparib (Talzenna^®^) ○ used for ovarian, peritoneal, breast, and fallopian tube cancers PI3K inhibitors: idelalisib (Zydelig^®^), alpelisib (Piqray^®^), duvelisib (Copiktra^®^), copanlisib (Aliqopa^®^) ○ used for breast cancer, lymphomas, and chronic lymphocytic leukemia mTOR inhibitors: everolimus (Afinitor^®^), temsirolimus (Torisel^®^), sirolimus (Rapamune^®^) ○ used for breast and pancreatic cancers, carcinomas (neuroendocrine and renal cell), and others Immune checkpoint inhibitors: CTLA-4 inhibitor—ipilimumab (Yervoy^®^), PD-1 inhibitors—Nivolumab (Opdivo^®^), Pembrolizumab (Keytruda^®^), Cemiplimab (Libtayo^®^), PD-L1 inhibitors—Avelumab (Bavencio^®^), durvalumab (Imfinzi^®^), atezolizumab (Tecentriq^®^) ○ used for various cancers including melanoma, lymphomas, carcinomas, and breast, cervical, colorectal, gastric, endometrial, and bladder cancers AR antagonists: abiraterone acetate (Zytiga^®^), megestrol acetate (Megace^®^) ○ primarily used for androgen-dependent prostate cancers Nonsteroidal antiandrogens: bicalutamide (Casodex^®^), flutamide (Eulexin^®^), nilutamide (Nilandron^®^), apalutamide (Erleada^®^), enzalutamide (Xtandi^®^) ○ primarily used for androgen-dependent prostate cancers TKIs: imatinib (Gleevec^®^), dasatinib (Sprycel^®^), nilotinib (Tasigna^®^), erlotinib (Tarceva^®^), sorafenib (Nexavar^®^), lapatinib (Tykerb^®^) ○ used for various cancers including chronic myeloid leukemia, non-small cell lung cancer, gastrointestinal stromal tumors; also breast, colorectal, medullary thyroid, and prostate cancers, and hepatocellular and renal cell carcinomas Angiogenesis inhibitors: direct VEGF inhibitors—aflibercept (Eylea^®^), ramucirumab (Cyramza^®^), bevacizumab (Avastin^®^), VEGF-TKIs—cabozantinib (Cabometyx^®^), regorafenib (Stivarga^®^), sorafenib (Nexavar^®^), sunitinib malate (Sutent^®^) ○ used for colorectal, stomach, thyroid, kidney, liver, and other cancers		

**Table 3:** Select miRNA molecules and siRNA/shRNA molecular targets described in current preclinical studies on TNBC [60,61,62,63,64,71,76,77,78,79,80,81,82,83,84,85,86,87,88,89,90,91,92,93,94,95,96,97,98,99,100,101,102].

**miRNA Name**	**miRNA Class**	**Key Function(s)**	**Reference(s)**
miR-143	Oncosuppressor	Suppression of cell proliferation, migration, and glycolytic pathway; apoptosis induction; tumor growth inhibition Target gene(s): *MUC1* [71]; *HK2* [76]	[76]
miR-127	Oncosuppressor	Suppression of cell proliferation, migration, and invasion Target gene(s): *CERK*, *NANOS1*, *FOXO6*, *SOX11*, *SOX12*, *FASN*, *SUSD2*	[77]
miR-218-5p	Oncogenic	Promotion of cell proliferation, bone metastasis, and tumor growth in the bone marrow; osteoclast activation and bone resorption; mediating acquisition of osteomimetic properties of bone metastatic BCa cellsTarget gene(s): *SOST*, *SFRP2*	[78]
miR-127-5p	Oncosuppressor	Mediation of M1 macrophage polarization Target gene(s): *CXCR4*	[79]
miR-34a	Oncosuppressor	Regulation of *eEF2K* gene; inhibition of tumor cell proliferation and growth, cell migration, and invasion Target gene(s): *NOTCH1*, *BCL2*, *CD44*, *SIRT1*, *RAC1*, *FOSL1* (Fra-1) [63]; *TP53*, *NOTCH2* [74]; *EEF2K*, *FOXM1* [80]	[63,80]
miR-335	Oncosuppressor	Inhibition of tumor re-initiation, cell growth and proliferation, and tumor metastasis Target gene(s): *SOX4*	[81]
miR-159	Oncosuppressor	Inhibition of cell proliferation and decrease in overall BCa incidence and progression Target gene(s): *TCF7*	[62]
miR-542-3p	Oncosuppressor	Tumor suppression; regulator of p53 tumor suppressor and anti-apoptotic protein survivin; apoptosis promotion Target gene(s): *TP53*, *BIRC5*	[64]
miR-603	Oncosuppressor	Tumor suppression; cell proliferation, migration/invasion, and tumorigenesis through regulation of *eEF2K* gene Target gene(s): *EEF2K*	[82]
miR-200 family	Oncosuppressor	Inhibition of tube formation ability through *PDGFR*β gene repression; regulation of tumor-mediated vasculogenesis and EMT; inhibition of tumor proliferation and metastasis; suppression of cell migration, invasion, and stemness; overcoming resistance to standard therapies Target gene(s): *PRKCA* [74]; *ZEB1/2*, *SNAI1/2* [71,74]; *JAG1*, *MAML2/3*, *PIK3CA* [70]; *PDGFRB* [83]	[83]
miR-9	Oncogenic	EMT progression; formation of vascular-like structures Target genes(s): *CHN1* [70,71]; *PDGFRB* [71,83]	[83]
miR-134	Oncosuppressor	Inhibition of cell migration and invasion; direct regulator of *STAT5B* gene and indirectly of Hsp90 Target gene(s): *STAT5B*	[84]
miR-708	Oncosuppressor	Inhibition of tumor cell migration and metastasis Target gene(s): *NNAT*	[85]
miR-125b	Oncogenic	Promotion of cell proliferation and tumorigenesis; fibroblast activation through suppression of *TP53* gene Target gene(s): *TP53*, *TP53INP1*	[86]
miR-21	Oncogenic	Antiapoptotic activity, tumor proliferation, and drug resistance; enhancing migration and invasion Target gene(s): *HIF1A (HIF1α)*, *TIMP3*, *TM1* [70]; *PDCD4*, *PTEN* [70,71]; *TPM1*, *TGFBR2* [71]	[60]
**siRNA/shRNA Molecular Genetic Targets**			
siRNA targets: *ADAM9* [87]; *AKT2* [88]; *EGFR* [89]; *CD44* [90]; *BRD4* [91]; *IKBKE* [61]; *ICAM1* [92]; *TTK*, *CDC20* [93]; *POLR2A* [94]; survivin [64]; *MTOR* (mTORC2) [95]; oncogenic lncRNAs TMPO-AS1 [96]/DANCR [97]; *CD44* [98]; *EIF4E* (eIF4E) [99]; *ZRANB1* [100] shRNA targets: *EZH2* [100]; *LGALS1* (galectin-1) [101]; *USP39* [102]			

**Table 4:** Clinical studies using RNA-based therapeutics for targeted treatment of TNBC (reference: clinicaltrials.gov, 11 October 2021).

Study Name and Clinical Trial Identifier	Sponsor/Collaborator	Intervention/Treatment Method	Study Recruitment Status
NCT03739931: Dose Escalation Study of mRNA-2752 for Intratumoral Injection to Participants With Advanced Malignancies	ModernaTX, Inc./AstraZeneca	Biological: mRNA-2752 Biological: Durvalumab (MEDI4736)	Recruiting; Phase 1
NCT02316457: RNA-Immunotherapy of IVAC_W_bre1_uID and IVAC_M_uID (TNBC-MERIT)	BioNTech SE/Seventh Framework Programme	Biological: IVAC_W_bre1_uID Biological: IVAC_W_bre1_uID/IVAC_M_uID	Active, not recruiting; Phase 1
NCT01837602: cMet CAR RNA T Cells Targeting Breast Cancer	University of Pennsylvania	Biological: c-Met RNA CAR-T cells	Completed; Phase 1
NCT04504669: First Time in Human Study of AZD8701 With or Without Durvalumab in Participants With Advanced Solid Tumors	AstraZeneca	Drug: AZD8701 Biological: Durvalumab (MEDI4736)	Recruiting; Phase 1

**Table 5:** Overview of major RNA-based therapeutic methods, main delivery mechanisms, and perceived advantages and disadvantages [34,107,110,111,112,113,114,115,116,117,118,119,120].

Therapeutic/Prophylactic Approach		Delivery Method(s)	Advantages	Disadvantages
RNAi	miRNA	-LNP delivery -Biopolymers -Covalent conjugation -Liposome -Polymeric, carbon, silica, and gold NPs -Dendrimer and micelle systems -Naked delivery -CPPs -Nanoplexes	-Dual mimetic/agonistic and replacement/antagonistic functions -Chemically synthesized and readily chemically modifiable -Less immunogenic than proteins -Small size	-Unstable -Nuclease-mediated degradation -Off-target effects -Not yet developed in clinical trials -More technologically challenging -Complementarity to target mRNA is not exact; lack of specificity
	siRNA		-More stable than miRNA -Easily and rapidly generated -Chemically synthesized and readily chemically modifiable -Less immunogenic than proteins -Small size -Already developed in clinical trials -100% complementarity to target mRNA—can knock down specific genes -High transfection efficiency	-Potential minor off-target exceptions and transient effects -Induction of nonspecific immune responses
Vaccine immunotherapy	mRNA vaccine	-Encapsulated LNP delivery-Naked delivery -Cationic liposomes -Cationic nanoemulsions -Dendrimer nanoparticles -Protamine liposome -Polysaccharide particle -Electroporation -Cationic polymer	-Thermally stable -Synthetic production (egg and cell-free) -Rapid and scalable production -Non-infectious, non-integrating, and naturally degraded -Expression in situ to produce antigens with structure unaltered by in vivo manufacturing process	-Concerns with instability (particularly unstable in plasma) -Limited immunogenicity data in humans -Potential toxic effect of free extracellular mRNA -Inflammation due to enhanced type I IFN activation
	DC (cell-based) vaccine	-Direct infusion of DCs through subcutaneous, intradermal, intranodal, intralymphatic, or intravenous routes	-High immunogenicity -Control of antigen presentation	-Expensive and difficult to produce -Vascular injury/electrolyte imbalances may occur after leukapheresis
	Gene-based vaccine (DNA/RNA)	-Lipid and polymeric micro- and nanoparticles -Cationic liposomes -Microspheres -Liposome-derived nanovesicles	-Easy delivery of multiple antigens -Induction of adaptive, and humoral immunity (B and T cell immune responses) -Stimulation of innate immune response -Non-infectious -Egg and cell-free -Not restricted to HLA-patient type -Rapid and scalable production -DNA vaccines: chemically stable -RNA vaccines: non-integrating, natural degradation	-Specific transportation/storage conditions for RNA vaccines -Both vaccines poorly immunogenic in humans -Potential integration of DNA vaccines into human genome -Chemical instability of RNA vaccines
	Viral vectored vaccines (oncolytic virotherapy)	-Liposomal, polymeric, or nanoparticle (magnetic and metallic NPs) delivery through intravenous, intratumoral, or intraperitoneal routes -Cell carriers	-Induction of adaptive, and humoral immunity; stimulation of innate immune response -High immunogenicity -Easy to produce on a large scale	-Induction of anti-vector immunity -Cell-based manufacturing -Potential high toxicity -Risk of undesired infections
Combination therapy	RNAi/small-molecule chemotherapeutics	-Nanoparticles (gold and mesoporous silica) -Cationic liposome -Micelle system -Dendrimer/supramolecular systems	-Overcome multidrug resistance -Produce additive or synergistic anti-cancer effects (promote apoptosis and autophagy) -Reduce drug-related toxicities -Increased spectrum of activity -Revert EMT -Suppress tumor angiogenesis -Downregulate MDR proteins, including ABC transporters and P-gp	-Potential risk for development of novel adverse reactions/increased toxicity -Increased risk for unfavorable interactions -Increased cost -Antagonistic effects
